# Adherence and perceived barriers to oral antiviral therapy for chronic hepatitis B

**DOI:** 10.1080/16549716.2018.1433987

**Published:** 2018-02-15

**Authors:** Kerui Xu, Li-Ming Liu, Paraskevi A. Farazi, Hongmei Wang, Fedja A Rochling, Shinobu Watanabe-Galloway, Jian-Jun Zhang

**Affiliations:** ^a^ Department of Epidemiology, College of Public Health, University of Nebraska Medical Center, Omaha, NE, USA; ^b^ Department of Hepatology, Hubei Third People’s Hospital, Wuhan, Hubei, China; ^c^ Department of Health Services Research & Administration, College of Public Health, University of Nebraska Medical Center, Omaha, NE, USA; ^d^ Division of Gastroenterology and Hepatology, Department of Internal Medicine, College of Medicine, University of Nebraska Medical Center, Omaha, NE, USA

**Keywords:** China, chronic hepatitis B, nucleot(s)ide analogs, medication adherence, Morisky Medication Adherence Scale

## Abstract

**Background**: Globally, of the 248 million people chronically infected with the hepatitis B virus (HBV), 74 million reside in China. Five oral nucleot(s)ide analogs (NUCs) have been approved for the treatment of chronic hepatitis B (CHB) in China.

**Objectives**: The aims of this study were to determine rates of adherence to NUC therapy in patients with CHB, to identify the self-perceived barriers to adherence, and to examine the factors associated with adherence.

**Methods**: Questionnaire-based interviews were administered among Chinese patients with CHB at hepatology clinics of a tertiary hospital in the city of Wuhan, China. Adults aged 18 years or older prescribed with NUCs were recruited and interviewed to complete a 27-item questionnaire in a private setting, and adherence was measured using the Morisky Medication Adherence Scale (MMAS-8).

**Results**: Among 369 participants, only 16.5% had high adherence (score of 8), 32.2% had medium adherence (score of 6 to <8), and 51.2% were measured with low adherence (score of <6). A logistic regression model was used to determine the factors associated with medication adherence. Significant predictors of high adherence consisted of urban residency, non-cirrhotic status, not using prescribed pills other than HBV medications, and reminders from family members. The five most common reasons for skipping NUCs were that medication(s) are expensive (48.7%), forgetfulness (45.1%), have experienced or worry about potential side effects (19.8%), do not want others to know about my medication(s) usage (18.5%), and ran out of pills and do not have time to refill (15.9%).

**Conclusions**: This study revealed that adherence rates to oral antiviral therapy were far from optimal. This finding should generate public attention, and it would be beneficial for interventional programs to target Chinese patients from rural regions, as well as patients with low socioeconomic status, cirrhosis, and taking multiple medications.

## Background

Hepatitis B virus (HBV) infection is endemic in China. Globally, China is among the countries with a high prevalence of HBV infection. Of the 248 million people who are chronically infected with HBV worldwide, approximately 74 million reside in China [1]. The biggest health concerns of HBV infection are risks associated with chronic hepatitis, including cirrhosis, liver failure and hepatocellular carcinoma (HCC) [2]. It is estimated that 85% of HCC cases in China are HBV-related [3], and China accounts for half of the total number of liver cancer cases and deaths worldwide [4]. Five oral nucleot(s)ide analogs (NUCs), conventional interferon alpha (IFN-α), and two formulations of pegylated interferon alpha (PEG-IFNα) have been approved for treating chronic hepatitis B (CHB) infection in China [5]. Based on guidelines established by the Chinese Society of Hepatology and Chinese Society of Infectious Diseases, all NUCs are recommended as first-choice treatments [6]. On the other hand, according to the American Association for the Study of Liver Disease (AASLD) and several international guidelines, entecavir and tenofovir are recommended as the first-line of NUC therapy in the treatment of CHB [7,8]. Although randomized clinical trials have demonstrated that entecavir and tenofovir have low incidence of drug resistance and a potent antiviral effect [5], due to the high costs of these medications and inadequate medical insurance coverage, entecavir and tenofovir are not affordable or reimbursable for many Chinese patients. Therefore, low-to-moderate generic barrier drugs, including lamivudine, telbivudine, and adefovir dipivoxil are still commonly used in China [9,10].

Adherence to antiviral therapy is fundamental for the clinical management of patients with CHB [11,12]. Long-term viral suppression was found to be associated with histologic improvement in the reduction of fibrosis and ultimately regression of cirrhosis [13]. Furthermore, a study has demonstrated that adult patients with CHB need over 2 years of NUC treatment to reduce risk of cirrhosis, HCC or CHB-related death [14]. In order to achieve and maintain virologic suppression, avoid virologic breakthrough, and attain undetectable levels of HBV DNA, optimal medication adherence is essential [15]. Antiviral therapy functions to prevent, delay, and reverse disease progression, leading to improved disease management and ultimately result in better survival [16].

A limited number of studies have utilized questionnaires to investigate the adherence to HBV antiviral therapy and factors associated with adherence [17–19]; however, these studies were limited to small sample sizes. Additionally, there is a lack of research focused to assess the self-perceived barriers and facilitators for adherence to HBV antiviral therapy. Since China has a high prevalence of CHB infection, it is crucial to understand the adherence and obstacles for HBV treatment using a validated instrument. Findings generated from this study could potentially be utilized in developing strategic preventive measures to improve medication adherence in regions of the world with a high prevalence of HBV infection. The aims of this study were to (i) determine rates of adherence to NUC antiviral therapy using the Morisky Medication Adherence Scale in Chinese patients with CHB, (ii) identify the self-perceived barriers to NUC adherence, and (iii) investigate the impact of sociodemographic and clinical factors, as well as treatment-related factors and perceptions of disease on NUC adherence.

## Methods

### Study design and data collection

This cross-sectional study was conducted from February to May 2017 at the Department of Hepatology of Hubei Third People’s Hospital, Wuhan, China. The Hubei Third People’s Hospital is a large tertiary hospital with areas in medicine, research, teaching, prevention and rehabilitation. It serves as the national base of standardized residency training and the national base of clinical trials for drug development. The study utilized a structured questionnaire, which was designed based on the opinions’ from experts in hepatology and previous studies on HBV medication adherence [17,18]. The source population comprised CHB patients who were prescribed with one or more NUCs, and eligible participants consisted of adults aged 18 years or older. Patients co-infected with hepatitis C, hepatitis D or human immunodeficiency virus, pregnant patients, and patients prescribed with NUCs less than 3 months ago were excluded. A pilot test of 30 patients was conducted to determine the feasibility and suitability of the questionnaire, and adjustments of the questionnaire were made accordingly. An interview-based, rather than a self-administered questionnaire was conducted to reduce the likelihood of participants skipping questions. Informed consent was obtained from all participants and in-person interviews were conducted in a private setting by a hepatologist in the hepatology clinics.

### Measures and assessment

The questionnaire comprised four sections. Section I consisted of 9 multiple-choice and fill-in-the-blank questions concerning basic sociodemographic and clinical information. The factors of interest included age, gender, current region of residence, education level, annual household income, medical insurance status, duration of known HBV infection, liver cirrhosis status, and the presence of other chronic diseases. In China, three main types of social medical insurance programs have been established: *Urban Employee’s Basic Medical Insurance* (UEBMI) works to cover insurance for the urban working population, *Urban Resident Basic Medical Insurance* (URBMI) provides care for the unemployed urban residents, and *New Rural Cooperative Medical System* (NCMS) provides financial subsidies for residents from rural regions [20].

Medication adherence was assessed by the 8-item Morisky Medication Adherence Scale (MMAS-8) in Section II of the questionnaire [21]. The MMAS-8 is a simple, reliable, and widely used instrument for determining adherence to prescribed medications [22]. The MMAS-8 has been demonstrated to be useful in identifying low adherence in clinical settings [23]. Moreover, a previous study has utilized the MMAS-8 to examine adherence to HBV treatment [24]. The Morisky Scale is comprised eight questions (score range: 0–8), with each item measuring a specific medication adherence behavior. The first seven items are Yes-or-No questions and the last item has five options. Adherence levels of high, medium, and low are defined with MMAS-8 scores of 8 points, 6 to <8 points, and <6 points, respectively. The validated Chinese translation was provided by Prof. Donald E. Morisky, as well as permission to use this scale. In Section III of the questionnaire, patients with moderate or low adherence were asked to choose the barrier(s) for taking NUCs or reason(s) for skipping NUCs.

The last section comprised 10 questions concerning treatment regimen and patient perceptions. Treatment-related questions consisted of type of NUC taken, duration of current antiviral therapy, use of other medications for treating HBV, number of other prescribed pills taken daily (exclude all medications used for HBV treatment), follow-up regularly at the clinic, understanding the physicians’ recommendations, use of memory aids (e.g. clock alarm, phone alarm), and reminders from family members. In addition, participants were interviewed about their perceptions of disease condition and current health condition in general.

### Statistical analysis

Data collected were coded and analyzed using SAS 9.4 (SAS Institute Inc., Cary, NC). A value of *P *< .05 in a two-tailed test was considered statistically significant. Descriptive statistics were performed, and variables were expressed as frequencies and percentages. The association of sociodemographic and clinical factors, as well as treatment-related factors and perceptions of disease with medication adherence levels were examined using χ^2^ test or Fisher Exact test. A multinominal logistic regression model with stepwise model selection (inclusion with *P *< .10) using ‘high adherence’ group as reference category was built to determine the independent predictors for medication adherence, and all factors (listed in Tables 1 and 2) were inserted into the model. The adjusted odds ratio (OR) and 95% confidence intervals (CI) were generated in the final model.

**Table 1.** A comparison of adherence to HBV antiviral therapy by patient sociodemographic and clinical characteristics (*n* = 369).

Characteristics	High Adherence(*n *= 61) *n* (%)	Medium Adherence(*n *= 119) *n* (%)	Low Adherence(*n *= 189) *n* (%)	Total Count(*n *= 369)	*P* Value
***Age group (years)***					.07
18**–**39	14 (15.2)	34 (37.0)	44 (47.8)	92	
40**–**49	21 (20.6)	39 (38.2)	42 (41.2)	102	
50**–**59	11 (13.6)	26 (32.1)	44 (54.3)	81	
≥60	15 (16.0)	20 (21.3)	59 (62.8)	94	
***Gender***					.51
Male	36 (14.9)	78 (32.4)	127 (52.7)	241	
Female	25 (19.5)	41 (32.0)	62 (48.4)	128	
***Region of residence***					**<.001**
Urban	55 (18.5)	110 (36.9)	133 (44.6)	298	
Rural	6 (8.5)	9 (12.7)	56 (78.9)	71	
***Education level***					**<.001**
Middle school or below	9 (11.4)	18 (22.8)	52 (65.8)	79	
High School	32 (16.4)	49 (25.1)	114 (58.5)	195	
College or above	20 (21.1)	52 (54.7)	23 (24.2)	95	
***Household income (RMB)***					.**003**
<50K	15 (15.0)	21 (21.0)	64 (64.0)	100	
50K**–**80K	29 (15.8)	59 (32.1)	96 (52.2)	184	
80K**–**150K	13 (19.7)	28 (42.4)	25 (37.9)	66	
>150K	4 (21.1)	11 (57.9)	4 (21.1)	19	
***Type of insurance****^¶^*					**<.001**
UEBMI	51 (20.2)	89 (35.2)	113 (44.7)	253	
URBMI	3 (13.0)	8 (34.8)	12 (52.2)	23	
NCMS	3 (4.7)	8 (12.5)	53 (82.8)	64	
OOP	2 (18.2)	3 (27.3)	6 (54.6)	11	
Others	2 (11.1)	11 (61.1)	5 (27.8)	18	
***Duration of HBV infection (years)***					.06
0**–**5	20 (18.9)	36 (34.0)	50 (47.2)	106	
6**–**15	22 (15.2)	56 (38.6)	67 (46.2)	145	
>15	19 (16.1)	27 (22.9)	72 (61.0)	118	
***Cirrhosis status***					**<.001**
Yes	9 (7.6)	25 (21.2)	84 (71.2)	118	
No	52 (20.7)	94 (37.5)	105 (41.8)	251	
***Other chronic diseases***					0.12
0	39 (17.4)	82 (36.6)	103 (46.0)	224	
1	13 (13.5)	24 (25.0)	59 (61.5)	96	
≥2	9 (18.4)	13 (26.5)	27 (55.1)	49	

*^¶^*Abbreviation: UEBMI, Urban Employee’s Basic Medical Insurance; URBMI, Urban Resident Basic Medical Insurance, NCMS, New Rural Cooperative Medical System; OOP, out-of-pocket.

## Results

### Patient characteristics

A total of 369 valid questionnaires were collected, with a response rate of 92.5%. The mean age of the participants was 49.1 ± 13.3 years, and the average duration of known HBV infection and duration of current antiviral therapy were 12.7 ± 9.4 years and 64.5 ± 55.4 months, respectively. The majority of patients were males (65.3%), resided in urban regions (80.8%), graduated with a highest degree from high school (52.9%), had annual household income of 80K RMB (USD ~$12.7K) or lower (77.0%), had the UEBMI medical insurance (68.6%), were non-cirrhotic (68.0%), and did not present other chronic diseases (60.7%) (Table 1). A variety of treatment regimens were prescribed, with 337 patients on NUC monotherapy and 32 patients on NUC combination therapy. The majority of patients received entecavir (n = 224, 60.7%), followed by adefovir (n = 100, 27.1%), lamivudine (n = 45, 12.2%), telbivudine (n = 31, 8.4%) and tenofovir (n = 1, 0.3%). In addition, 28 patients (7.6%) received entecavir plus adefovir, and 4 patients (1.1%) received lamivudine plus adefovir.

### Medication adherence rates

Adherence rates were determined using the Morisky Medication Adherence Scale (Appendix). Based on the MMAS-8 scoring system, a total of 61 patients (16.5%) had high adherence, 119 patients (32.2%) exhibited medium adherence, and 189 patients (51.2%) were measured with low adherence. A further analysis of the MMAS-8 data showed that overall, 41.2% of patients stated that they sometimes forget to take medication(s), and 34.7% reported of having missed taking medication(s) sometime within the past 2 weeks. A number of patients (15.2%) reported to have cut back or stopped taking medication(s) without telling their doctors because they felt worse, or because they felt the symptoms were under control (19.8%). Moreover, 89 patients (24.1%) reported of sometimes forgetting to bring along medication(s) when traveling or leaving home. The vast majority of patients (94.6%) took their medication(s) yesterday, but most patients (52.3%) felt that it was a hassle to stick with their current treatment plan. When asked about ‘how often do you have difficulty remembering to take all your antiviral medication(s)?’, 30.9% responded never/rarely, 27.1% once a while, 31.2% sometimes, 7.9% usually, and 3.0% all the time.

### Factors associated with medication adherence

The association of patient sociodemographic and clinical factors with medication adherence was generated from univariate analysis. As shown in Table 1, adherence was significantly associated with region of residence (*P* < .001), education level (*P* < .001), annual household income (*P* = .003), type of medical insurance (*P* < .001) and cirrhosis status (*P* < .001). Patients with education level of college or above, annual household income of greater than 150K RMB (USD ~$23.7K), UEBMI medical insurance, as well as patients without cirrhosis and resided in urban regions were more likely to have high adherence. Table 2 presents the association of treatment-related factors and perceptions of disease with medication adherence. Adherence was significantly associated with duration of current antiviral therapy (*P* = .02), number of other prescribed pills taken daily (*P* = .016), follow up regularly at the clinic (*P* = .016), reminders from family members (*P* = .049), and perception of current health condition (*P* = .021). As shown, patients with a shorter duration of current treatment at 0**–**24 months, not using other prescribed pills, followed up regularly at the clinic, received reminders from family members, and perceived their current health condition to be very good were more likely to have high adherence.

**Table 2.** A comparison of adherence to HBV antiviral therapy by treatment-related characteristics and perceptions of disease (*n* = 369).

Characteristics	High Adherence(*n *= 61) *n* (%)	Medium Adherence(*n *= 119) *n* (%)	Low Adherence(*n *= 189) *n* (%)	Total Count(*n *= 369)	*P* Value
***Duration of current therapy (months)***					.**003**
0**–**24	21 (27.6)	26 (34.2)	29 (38.2)	76	
25**–**60	23 (13.3)	64 (37.0)	86 (49.7)	173	
>60	17 (14.2)	29 (24.2)	74 (61.7)	120	
***Use of other medications to treat HBV***					.64
Yes, Chinese medicine	20 (14.4)	41 (29.5)	78 (56.1)	139	
Yes, Western medicine	2 (13.3)	6 (40.0)	7 (46.7)	15	
No	39 (18.1)	72 (33.5)	104 (48.4)	215	
***Number of prescribed pills taken daily^¶^***					.**002**
0	49 (19.8)	89 (36.0)	109 (44.1)	247	
1	8 (13.1)	14 (23.0)	39 (63.9)	61	
≥2	4 (6.6)	16 (26.2)	41 (67.2)	61	
***Regularly visit clinic for HBV***					.**016**
Yes	55 (17.5)	108 (34.4)	151 (48.1)	314	
No	6 (10.9)	11 (20.0)	38 (69.1)	55	
***Understand what the physicians*** ***recommend***					.62
Yes	56 (17.1)	106 (32.4)	165 (50.5)	327	
No	5 (11.9)	13 (31.0)	24 (57.1)	42	
***Use of memory aids***					.28
Yes	4 (10.8)	16 (43.2)	17 (46.0)	37	
No	57 (17.2)	103 (31.0)	172 (51.8)	332	
***Reminders from family members***					.**049**
Yes	28 (21.7)	45 (34.9)	56 (43.4)	129	
No	33 (13.8)	74 (30.8)	133 (55.4)	240	
***Perception of disease condition***^δ^					.12
Severe	6 (16.7)	9 (25.0)	21 (58.3)	36	
Moderate	24 (15.7)	41 (26.8)	88 (57.5)	153	
Mild	26 (16.5)	62 (39.2)	70 (44.3)	158	
Don’t know	5 (22.7)	7 (31.8)	10 (45.5)	22	
***Perception of current health condition***					.**021**
Very good	5 (45.5)	3 (27.3)	3 (27.3)	11	
Good	20 (17.5)	43 (37.7)	51 (44.7)	114	
Fair	30 (14.6)	67 (32.5)	109 (52.9)	206	
Poor	6 (15.8)	6 (15.8)	26 (68.4)	38	

*^¶^*HBV medications are not included.

^δ^Participants who answered ‘don’t know’ are excluded from analysis.

### Predictors of medication adherence

Table 3 illustrates the results from logistic regression analysis on the determinants of medication adherence. Region of residence, cirrhosis status, number of other prescribed pills taken daily, and reminders from family members were significant predictors of adherence to NUCs. Patients residing in urban regions were 4.88 times (OR: 0.21; 95% CI: 0.07–0.57; *P* = .002) more likely of having high adherence as opposed to low adherence compared to patients from rural regions. Likewise, cirrhotic patients were 3.17 times (OR: 3.17; 95% CI: 1.26–7.95; *P* = .014) more likely to have low adherence than high adherence compared to patients without cirrhosis. Patients receiving reminders from family members were 3.13 times (OR: 0.32; 95% CI: 0.16–0.66; *P* = .002) more likely to belong to the high adherence group. Additionally, compared to patients not using other prescribed pills, those taking 2 or more other prescribed pills daily were more likely to report medium (OR: 4.64; 95% CI: 1.09–19.80; *P* = .038) or low adherence (OR: 5.45; 95% CI: 1.36–21.81; *P* = .017) as opposed to high adherence.

**Table 3.** Multinomial logistic regression of the effect of sociodemographic characteristics, clinical characteristics, treatment-related characteristics and perceptions of disease on adherence to HBV antiviral therapy (*n* = 369).

	Medium Adherence versus High Adherence		Low Adherence versus High Adherence	
Characteristics	OR (95% CI)	*P* Value	OR (95% CI)	*P* Value
***Region of residence***				
Rural	Reference		Reference	
Urban	1.27 (0.38, 4.20)	.70	0.21 (0.07, 0.57)	.**002**
***Cirrhosis status***				
No	Reference		Reference	
Yes	1.33 (0.48, 3.65)	.58	3.17 (1.26, 7.95)	.**014**
***Number of prescribed pills taken daily^¶^***				
0	Reference		Reference	
1	1.12 (0.38, 3.30)	.83	2.23 (0.84, 5.93)	.11
≥2	4.64 (1.09, 19.80)	.**038**	5.45 (1.36, 21.81)	.**017**
***Reminders from family members***				
No	Reference		Reference	
Yes	0.69 (0.34, 1.41)	.31	0.32 (0.16, 0.66)	.**002**

All factors from Tables 1 and 2 were examined in stepwise model selection.

*^¶^* HBV medications are not included.

### Perceived barriers toward medication adherence

The frequencies of self-perceived barriers were analyzed and are described in Table 4. The top five reasons for skipping NUCs were that ‘Medication(s) are expensive and difficult to afford’ (48.7%), ‘Forgetfulness’ (45.1%), ‘Have experienced or worry about potential side effects’ (19.8%), ‘Do not want others to know about my medication(s) usage’ (18.5%), and ‘Ran out of pills and do not have time to refill’ (15.9%).

**Table 4.** Perceived barriers toward compliance to HBV antiviral therapy among patients with medium and low adherence in rank order (*n* = 308).

Barriers	*n* (%)
(1) Medication(s) are expensive and difficult to afford	150 (48.7)
(2) Forgetfulness	139 (45.1)
(3) Have experienced or worry about potential side effects	61 (19.8)
(4) Do not want others to know about my medication(s) usage	57 (18.5)
(5) Ran out of pills and do not have time to refill	49 (15.9)
(6) Feel better already and do not think it is necessary to continue	41 (13.3)
(7) Multiple medications are taken daily and cannot keep track of the dose for each	37 (12.0)
(8) Cannot tell the difference between taking/not taking medication(s)	33 (10.7)
(9) Insurance does not provide coverage when cost exceeds the limit	32 (10.4)
(10) Emotionally distressed about disease condition and have no desire to continue	15 (4.9)
(11) Physician did not inform me about the importance of taking medication(s) timely	7 (2.3)
(12) Difficulty swallowing	3 (1.0)

## Discussion

To our knowledge, this is the largest study utilizing self-report questionnaires to access the adherence and self-perceived barriers to HBV oral antiviral therapy. Based on our findings, adherence to NUCs among patients with CHB was found to be very poor, with 51.2% of patients reported to have low adherence. In comparison, a similar study that utilized structured questionnaires and conducted in Australia found that 74.1% of patients reported an adherence rate of 100% [18], while 66% and 52% of patients in similar studies conducted in the United States and the Netherlands were measured with an adherence rate of 90% [17,19]. In contrast, only 16.5% of patients from this study scored an adherence of 100%. Chotiyaputta et al. [17] observed that 73% of patients reported they did not miss a single dose of medication during the past 30 days, whereas we found that just 65.3% of patients did not miss taking medication(s) during the past 2 weeks. Furthermore, rates of high adherence reported in secondary studies that used pharmacy and medical records were also significantly higher than rate observed in our study [11,25–27].

It is widely known that major gaps in the economic development and health disparities exist between urban and rural regions in China [28]. Our findings demonstrated that urban residents were significantly more likely to have high adherence compared to residents from rural regions. In China, rural patients with CHB often have issues in accessing quality health services due to the lack of specialized clinical services established in rural areas [29]. The majority of quality hospitals are located in the urban regions with better trained healthcare professionals and more advanced technology [30]. Furthermore, HBV has been considered as an economically catastrophic disease, and costs of treatment are a major burden for rural patients [29]. An estimated figure has shown that less than 5% of rural patients are able to afford 1 year of treatment as opposed to 40% of patients in more developed regions [29]. In addition to issues concerning accessibility and costs of treatment, Chinese patients in rural regions also have poor health awareness; many infected-individuals are unaware of their HBV infection until symptoms appear [31,32].

The finding that cirrhotic patients were less adherent to NUCs may be explained by that patients who had better treatment adherence were less likely to develop cirrhosis. Furthermore, our results illustrated that patients taking 2 or more other prescribed pills on a daily basis were cited to be more likely of having low adherence. Four studies that investigated medication nonadherence among elderly populations prescribed with various medications have shown that a greater number of drugs was associated with worse adherence [33]. In a large-scale study, researchers examined the effect of previous prescription burden on adherence rates when antihypertensive or lipid-lowering therapy was added, and found that rates dropped to 41%, 30% and 20% among patients who received 0, 2, and 10 or more previous medications, respectively [34]. Polypharmacy is associated with nonadherence due to a number of factors, including regimen complexity, time and commitment, treatment costs, financial reimbursement, difficulty in managing co-existing illnesses, side effects, multiple prescribers, access to care, etc. [35].

Consistent with our findings, numerous studies have found a positive relationship between family support interventions and medication adherence [36]. A lack of family and social support has shown to be a predictive factor of nonadherence among patients treated for chronic illnesses [36], and research on the adherence to type II diabetes treatment demonstrated that family support was the strongest predictor of adherence [37]. Results generated from univariate analysis indicated that higher annual household income was significantly associated with better adherence, and Chotiyaputta et al. [17] also observed similar result. In contrast, while studies have observed that patients with poor adherence were more likely to be younger [17,19,27], an association between age and adherence was not found in this study, which may be explained by racial and ethnic differences in the study populations.

‘Medication(s) are expensive and difficult to afford’ was the most common reason for skipping NUCs. Over 60% of total patients in this study were prescribed with entecavir, which is known for its unaffordability to many Chinese patients [11]. Moreover, among the 182 patients who were prescribed with entecavir and were also measured with medium or low adherence, approximately half of them (50.5%) indicated ‘Medication(s) are expensive and difficult to afford’ and/or ‘Insurance does not provide coverage when cost exceeds the limit’ as the barrier for taking NUCs. Although entecavir has a higher cost, research has shown that entecavir is still more cost-effective than other NUCs [38]. One study evaluated the cost-effectiveness of NUCs in China, and assessed the thresholds at which the drugs would be cost-saving to the national treatment program [39]. The investigators found that generic entecavir would be the most cost-effective therapy unless the cost of tenofovir drops. Currently, several pharmaceutical companies in China are producing generic versions of entecavir, and the lowest reported price is at $1,258 (~7,955 RMB) per person-year or $105 (~664 RMB) per person-month [40]. Additionally, one study that estimated the cost of manufacturing generic entecavir at a minimum target price found that generic entecavir could actually be produced at $36 (~228 RMB) per person-year or $3 (~19 RMB) per person-month, which is substantially lower than the current price [40]. Since the patent for entecavir has expired, it would be cost-effective for the government to enter into negotiations with generic drug manufacturers of entecavir to obtain a less costly drug. Within a competitive market, an affordable and large-scale treatment system could provide immense health benefits to patients with CHB.

Another common barrier of medication adherence was ‘Forgetfulness’, which was cited as the main reason for skipping NUCs in the study conducted in Australia [18]. Forgetfulness can be partly dealt through providing reminders, such as from families and close friends, and the use of alarm clocks or automated text messages [41]. Nonetheless, forgetfulness can be influenced by cognitive factors [18], including a lack of awareness and knowledge concerning the health risks associated with disease condition. Therefore, healthcare providers should actively inform patients about the importance of adhering to antiviral therapy and the potential consequences for skipping NUCs. One study based on a review of medical records of 69 immigrant patients in Chicago discovered that concerns about the long-term safety of NUCs was cited as one of the main barriers to treatment initiation and one of the main reasons for treatment discontinuation [26]. Likewise in our study, ‘Have experienced or worry about potential side effects’ was identified as the third most common barrier to NUC adherence. This illustrates a misconception about NUCs; even though treatment of CHB can often be life-long, NUCs are generally safe and well-tolerated by patients [42]. Furthermore, a large number of patients cited ‘Do not want others to know about my medication(s) usage’ as a perceived barrier. In China, patients with CHB are living under a great amount of emotional distress and often face discrimination in life and work. Although HBV check-ups for employment and school enrollment have been banned since 2010, some employers still request job applicants to disclose HBV test results [43]. As a result, fear of disclosing HBV status may have negatively affected adherence to treatment. Therefore, with the help of psychiatrists and clinical psychologists, it would be beneficial to identify patients with psychological and emotional issues and offer the appropriate counseling, which would consist of alleviating emotional stress, overcoming fear, managing crises, and giving encouragements. Furthermore, ‘Ran out of pills and do not have time to refill’, ‘Feel better already and do not think it is necessary to continue’ and ‘Multiple medications are taken daily, difficult to track dose’ were other barriers that had an impact on NUC adherence. Nevertheless, these barriers were not cited as common reasons for skipping NUCs reported by Giang et al. [18].

This study is subject to certain limitations that should be addressed. One of the limitations is that because we utilized convenience sampling method, there is possibility that sampling bias was introduced and this may limit the external validity of the study’s results. In comparison to population-based probability sampling, convenience sampling produces estimates that are more generalizable to the sample studied, whereas results produced from population-based sampling could yield more representative estimates of the target population [44]. Furthermore, as a cross-sectional study, significant association between the factors of interest and outcome can be difficult to interpret, and causality cannot be established as correlation does not imply causation. For instance, non-cirrhotic status was a significant predictor of high adherence, but it is difficult to determine whether this was because better NUC adherence served to prevent adherent patients from developing cirrhosis, or that patients without cirrhosis tend to have better adherence. Additionally, a meta-analysis consisting of studies on NUC adherence indicated that studies rely on patient self-report may to subject to overestimation when compared to secondary studies using data from pill count and pharmacy refill claims [11]. The potential inflation in reporting may be a result of reporting bias. Last, since China is a culturally and economically diverse nation, findings generated from this study are subject to geographical limitations and should be taken into account when making application of the results in different parts of the world as well as in different regions of China.

## Conclusion

The finding of poor medication adherence among Chinese patients taking NUCs should generate public attention, and calls for healthcare providers to work collaboratively with researchers and community health leaders to develop more effective interventional methods to improve NUC adherence. These interventional programs should target patients from rural regions, patients with low socioeconomic status, cirrhotic patients, and patients prescribed with multiple medications. Additionally, patients with severe emotional distress or at risk for mental disorders should be identified and be provided with professional counseling to cope with social and psychological issues. Further studies should focus to investigate the efficacy and impact of medication adherence on viral suppression, and the rate of adherence needed to prevent antiviral resistance in Chinese patients with CHB.

## Paper context

This is the first known study conducted in China to examine the adherence and self-perceived barriers in HBV antiviral therapy. The findings demonstrated that adherence to antiviral therapy was very poor, with 51.2% participants measured to have low adherence. Interventional programs should target patients from rural regions, with low socioeconomic status, cirrhosis, and taking multiple medications. Furthermore, the government should enter into negotiations with the generic drug manufacturers of entecavir to make it more affordable.

## Appendix

### The 8-item Morisky Medication Adherence Scale (MMAS-8)

Use of the ©MMAS is protected by US and International copyright laws. Permission for use is required. A license agreement is available from: Donald E. Morisky, MMAS Research (MORISKY), 294 Lindura Court, Las Vegas, NV 89135-1415; dmorisky@gmail.com.

**Table:**

	Patient answer(Yes or No)
1. Do you sometimes forget to take your antiviral medication(s)?	
2. People sometimes miss taking their medication(s) for reasons other than forgetting. Thinking over the past two weeks, were there any days when you did not take your antiviral medication(s)?	
3. Have you ever cut back or stopped taking your antiviral medication(s) without telling your doctor because you felt worse when you took it?	
4. When you travel or leave home, do you sometimes forget to bring along your medication(s)?	
5. Did you take all your antiviral medication(s) yesterday?	
6. When you feel like your symptoms are under control, do you sometimes stop taking your medication(s)?	
7. Taking antiviral medication(s) every day is a real inconvenience for some people. Do you ever feel hassled about sticking to your treatment plan?	
8. How often do you have difficulty remembering to take all your medication(s)?	
A. Never/Rarely	
B. Once in a while	
C. Sometimes	
D. Usually	
E. All the time	

## References

[1] Schweitzer A, Horn J, Mikolajczyk RT, et al Estimations of worldwide prevalence of chronic hepatitis B virus infection: a systematic review of data published between 1965 and 2013. Lancet. 2015;386:1546–10. 26231459 10.1016/S0140-6736(15)61412-X

[2] El-Serag HB. Epidemiology of viral hepatitis and hepatocellular carcinoma. Gastroenterology. 2012;142(6):1264–1273.e1. 22537432 10.1053/j.gastro.2011.12.061 PMC3338949

[3] Song P, Gao J, Inagaki Y, et al. Biomarkers: evaluation of screening for and early diagnosis of hepatocellular carcinoma in japan and china. Liver Cancer. 2013;2(1):31–39. 24159594 10.1159/000346220 PMC3747538

[4] Torre LA, Bray F, Siegel R, et al Global cancer statistics, 2012. CA: Cancer J Clin. 2015;65:87–108. 25651787 10.3322/caac.21262

[5] Zheng X, Wang J, Yang D. Antiviral therapy for chornic hepatitis B in China. Med Microbiol Immunol. 2015;204:115–120. 25540038 10.1007/s00430-014-0380-z PMC4305090

[6] Chinese Society of Hepatology and Chinese Society of Infectious Diseases, Chinese Medical Association [The guideline of prevention and treatment for chronic hepatitis B (2010 version)]. Zhonghua Gan Zang Bing Za Zhi. 2011;19:13–24. 21272453 10.3760/cma.j.issn.1007-3418.2011.01.007

[7] Terrault NA, Bzowej NH, Chang K-M, et al. AASLD guidelines for treatment of chronic hepatitis B. Hepatology. 2016;63:261–283. 26566064 10.1002/hep.28156 PMC5987259

[8] Park JW, Kwak KM, Kim SE, et al Comparison of the long-term efficacy between entecavir and tenofovir in treatment- naïve chronic hepatitis B patients. BMC Gastroenterol. 2017;17:39. 28279168 10.1186/s12876-017-0596-7 PMC5345200

[9] Yu R, Fan R, Hou J Chronic hepatitis B virus infection: epidemiology, prevention, and treatment in China. Front Med. 2014;8:135–144. 24810645 10.1007/s11684-014-0331-5

[10] Chen EQ Current trends of combination therapy in chronic hepatitis B management in China. Arch of Hepat Res. 2015;1:1–4.

[11] Chotiyaputta W, Peterson C, Ditah FA, et al Persistence and adherence to nucleos(t)ide analogue treatment for chronic hepatitis B. J Heptaol. 2011;54:12–18. 10.1016/j.jhep.2010.06.016 20888661

[12] Ha NB, Garcia RT, Trinh HN, et al Medication nonadherence with long-term management of patients with hepatitis B e antigen-negative chronic hepatitis B. Dig Dis Sci. 2011;56:2423–2431. 21327918 10.1007/s10620-011-1610-5

[13] Chang TT, Liaw YF, Wu SS, et al Long-term entecavir therapy results in the reversal of fibrosis/cirrhosis and continued histological improvement in patients with chronic hepatitis B. Hepatology. 2010;52:886–893. 20683932 10.1002/hep.23785

[14] Zhang Q-Q, An X, Liu Y-H, et al. Long-term nucleos(t)ide analogues therapy for adults with chronic hepatitis B reduces the risk of long-term complications: a meta-analysis. Virol J. 2011;8:72. 21324130 10.1186/1743-422X-8-72 PMC3046930

[15] Zoulim F, Locarnini S Hepatitis B virus resistance to nucleos(t)ide analogues. Gastroenterology. 2009;137:1593–1608. 10.1053/j.gastro.2009.08.063 19737565

[16] Cooke GS, Main J, Thursz MR Treatment for hepatitis B. BMJ. 2010;340:b5429. 20051467 10.1136/bmj.b5429

[17] Chotiyaputta W, Hongthanakorn C, Oberhelman K, et al Adherence to nucleos(t)ide analogues for chronic hepatitis B in clinical practice virological breakthroughs. J Viral Hepat. 2012;19:205–212. 22329375 10.1111/j.1365-2893.2011.01494.x

[18] Giang L, Selinger CP, Lee AU Evaluation of adherence to oral antiviral hepatitis B treatment using structured questionnaires. World J Hepatol. 2012;4:43–49. 22400085 10.4254/wjh.v4.i2.43 PMC3295851

[19] Van Vlerken LG, Arends P, Lieveld FI, et al Real life adherence of chronic hepatitis B patients to entecavir treatment. Dig Liver Dis. 2015;47:577–583. 25936691 10.1016/j.dld.2015.03.024

[20] Wang S, Liu L, Li L, et al Comparison of Chinese inpatients with different types of medical insurance before and after the 2009 healthcare reform. BMC Health Serv Res. 2014;14:443. 25267508 10.1186/1472-6963-14-443 PMC4261594

[21] Morisky DE, Ang A, Krousel-Wood M, et al Predictive validity of a medication adherence measure in an outpatient setting. J Clin Hypertens (Greenwich). 2008;10:348–354. 18453793 10.1111/j.1751-7176.2008.07572.x PMC2562622

[22] Morisky DE, DiMatteo MR Improving the measurement of self-reported medication nonadherence: response to authors. J Clin Epidemiol. 2011;64:255–257. 21144706 10.1016/j.jclinepi.2010.09.002 PMC3109729

[23] Krousel-Wood M, Islam T, Webber LS, et al New medication adherence scale versus pharmacy fill rates in seniors with hypertension. Am J Manag Care. 2009;15:59–66. 19146365 PMC2728593

[24] Wojcik K, Piekarska A, Jalonowska E Adherence to antiviral therapy in HIV or HBV-infected patients. Przegl Epidemiol. 2016;70:27–32. 27344470

[25] Malespin M, Wong S, Siqueira F, et al Barriers to treatment of hepatitis B in an urban Chinatown community.J Clin Gastroenterol. 2012;46:e66–e70. 10.1097/MCG.0b013e31824e159c 22460162

[26] Allard N, Dev A, Dwyer J, et al Factors associated with poor adherence to antiviral treatment for hepatitis B. J Viral Hepat. 2017;24:53–58. 27502689 10.1111/jvh.12582

[27] Lieveld FI, van Vlerken LG, Siersema PD, et al Patient adherence to antiviral treatment for chronic hepatitis B and C: a systematic review. Ann Hepatol. 2013;12:380–391. 23619254

[28] Meng Q, Zhang J, Yan F, et al One country, two worlds – the health disparity in China. Glob Public Health. 2012;7:124–136. 21981140 10.1080/17441692.2011.616517

[29] Wallace J, Pitts M, Liu C, et al Needs assessment of viral hepatitis - China. Melbourne: Australian Research Centre in Sex, Health and Society, La Trobe University; 2015 Available from: http://www.nohep.org/wp-content/uploads/2016/07/Needs-Assessment-of-People-Living-with-Viral-Hepatitis-China.pdf

[30] Johnson J, Stoskopf C, Shi L Comparative health systems: a global perspective. Burlington: Jones & Bartlett Learning; 2017 p. 445.

[31] Wu W, Zhu Y, Yu C, et al Clinical features of treatment-naive patients with hepatitis B virus infection: A community-based survey from high- and intermediate-hepatitis B endemicity regions in Southeast China. Medicine (Baltimore). 2017;96:e6660. 28422873 10.1097/MD.0000000000006660 PMC5406089

[32] Xu K, Watanabe-Galloway S, Rochling FA, et al Practice, knowledge, and barriers for screening of hepatocellular carcinoma among high-risk Chinese patients. Ann Global Health. 2017;83:281–292. 10.1016/j.aogh.2017.02.002 PMC9001923 28619403

[33] Gellad WF, Grenard JL, Marcum ZA A systematic review of barriers to medication adherence in the elderly: looking beyond cost and regimen complexity. Am J Geriatr Pharmacother. 2011;9:11–23. 21459305 10.1016/j.amjopharm.2011.02.004 PMC3084587

[34] Benner JS, Chapman RH, Petrilla AA, et al Association between prescription burden and medication adherence in patients initiating antihypertensive and lipid-lowering therapy. Am J Health Syst Pharm. 2009;66:1471–1477. 19667004 10.2146/ajhp080238

[35] Marcum ZA, Gellad WF Medication adherence to multi-drug regimens. Clin Geriatr Med. 2012;28:287–300. 22500544 10.1016/j.cger.2012.01.008 PMC3335752

[36] Miller TA, DiMatteo MR Importance of family/social support and impact on adherence to diabetic therapy. Diabetes Metab Syndr Obes. 2013;6:421–426. 24232691 10.2147/DMSO.S36368 PMC3825688

[37] Glasgow RE, Toobert DJ Social environment and regimen adherence among type II diabetic patients.Diabetes Care. 1988;11:377–386. 3391088 10.2337/diacare.11.5.377

[38] Wang J Clinical utility of entecavir for chronic hepatitis B in Chinese patients. Drug Des Devel Ther. 2013;8:13–24. 10.2147/DDDT.S41423 PMC3865082 24376343

[39] Toy M, Hutton DW, So SK Cost-effectiveness and cost thresholds of generic and brand drugs in a national chronic hepatitis B treatment program in China. PLoS One. 2015;10:e0139876. 26536626 10.1371/journal.pone.0139876 PMC4633043

[40] Hill A, Gotham D, Cooke G, et al Analysis of minimum target prices for production of entecavir to treat hepatitis B in high- and low-income countries. J Virus Erad. 2015;1:103–110. 27482399 10.1016/S2055-6640(20)30484-2 PMC4946675

[41] Jimmy B, Jose J Patient medication adherence: measures in daily practice. Oman Med J. 2011;26:155–159. 22043406 10.5001/omj.2011.38 PMC3191684

[42] Kayaaslan B, Guner R Adverse effects of oral antiviral therapy in chronic hepatitis B. World J Hepatol. 2017;9:227–241. 28261380 10.4254/wjh.v9.i5.227 PMC5316843

[43] Yang T, Wu MC Discrimination against hepatitis B carriers in China. Lancet. 2011;378:1059. 21924982 10.1016/S0140-6736(11)61460-8

[44] Bornstein MH, Jager J, Putnick DL Sampling in developmental science: situations, shortcomings, solutions, and standards. Dev Rev. 2013;33:357–370. 25580049 10.1016/j.dr.2013.08.003 PMC4286359

